# Green Synthesis and Characterization of Silver Nanoparticles Using *Azadirachta indica* Seeds Extract: In Vitro and In Vivo Evaluation of Anti-Diabetic Activity

**DOI:** 10.3390/ph16121677

**Published:** 2023-12-01

**Authors:** Gauhar Rehman, Muhammad Umar, Nasrullah Shah, Muhammad Hamayun, Abid Ali, Waliullah Khan, Arif Khan, Sajjad Ahmad, Abdulwahed Fahad Alrefaei, Mikhlid H. Almutairi, Yong-Sun Moon, Sajid Ali

**Affiliations:** 1 Department of Zoology, Abdul Wali Khan University Mardan, Mardan 23200, Pakistan; umarm5030@gmail.com (M.U.); drabid@awkum.edu.pk (A.A.); arifkhanpak83@gmail.com (A.K.); sajjadmohmand891@gmail.com (S.A.); 2 Department of Chemistry, Abdul Wali Khan University Mardan, Mardan 23200, Pakistan; nasrullah@awkum.edu.pk (N.S.); drwali@awkum.edu.pk (W.K.); 3 Department of Botany, Abdul Wali Khan University Mardan, Mardan 23200, Pakistan; hamayun@awkum.edu.pk; 4 Department of Zoology, College of Science, King Saud University, Riyadh 11451, Saudi Arabia; afrefaei@ksu.edu.sa (A.F.A.); malmutari@ksu.edu.sa (M.H.A.); 5 Department of Horticulture and Life Science, Yeungnam University, Gyeongsan 38541, Republic of Korea; hangulmys@ynu.ac.kr

**Keywords:** green synthesis, AgNPs, *Azadirachta indica*, biosynthesize nanoparticles, diabetes, streptozotocin

## Abstract

Background: Diabetes mellitus (DM) is a non-communicable, life-threatening syndrome that is present all over the world. The use of eco-friendly, cost-effective, and green-synthesised nanoparticles as a medicinal therapy in the treatment of DM is an attractive option. Objective: In the present study, silver nanoparticles (AI-AgNPs) were biosynthesized through the green synthesis method using *Azadirachta indica* seed extract to evaluate their anti-diabetic potentials. Methods: These nanoparticles were characterized by using UV-visible spectroscopy, Fourier transform infrared spectrophotometers (FTIR), scanning electron microscopy (SEM), DLS, and X-ray diffraction (XRD). The biosynthesized AI-AgNPs and crude extracts of *Azadirachta indica* seeds were evaluated for anti-diabetic potentials using glucose adsorption assays, glucose uptake by yeast cells assays, and alpha-amylase inhibitory assays. Results: Al-AgNPs showed the highest activity (75 ± 1.528%), while crude extract showed (63 ± 2.5%) glucose uptake by yeast at 80 µg/mL. In the glucose adsorption assay, the highest activity of Al-AgNPs was 10.65 ± 1.58%, while crude extract showed 8.32 ± 0.258% at 30 mM, whereas in the alpha-amylase assay, Al-AgNPs exhibited the maximum activity of 73.85 ± 1.114% and crude extract 65.85 ± 2.101% at 100 µg/mL. The assay results of AI-AgNPs and crude showed substantial dose-dependent activities. Further, anti-diabetic potentials were also investigated in streptozotocin-induced diabetic mice. Mice were administered with AI-AgNPs (10 to 40 mg/kg b.w) for 30 days. Conclusions: The results showed a considerable drop in blood sugar levels, including pancreatic and liver cell regeneration, demonstrating that AI-AgNPs have strong anti-diabetic potential.

## 1. Introduction

Diabetes mellitus (DM) is a chronic illness due to inadequate insulin production by pancreatic β cells. Diabetes could be hereditary or acquired. The insufficiency of insulin is a consequence of high blood glucose levels, which cause damage to various body systems, especially the circulatory and nervous systems [1,2]. As evident from the data of the International Diabetes Federation (IDF), the incidence of diabetes is rising internationally. In 2021, 536.6 million people had diabetes worldwide, and by the year 2045, it is projected to increase by 46% to 783.2 million [3]. As previously estimated by the IDF and other surveys, almost 50% of all patients with diabetes are oblivious to their illness [4]. Changing one’s lifestyle to include increased physical exercise, consuming low-calorie foods, and avoiding inactive habits is necessary for DM prevention and control [5]. Although synthetic drugs like miglitol and acarbose have high inhibitory effects against alpha-amylase and alpha-glucosidase, they have consequences like causing diarrhea, nausea, vomiting, and intestinal swelling [6]. Therefore, plant extracts that are active in lowering serum glucose levels with slight or no side effects are used as hypoglycemic medications. Various compounds extracted from plants are used in combinational treatment for diabetes, such as *Azadirachta indica*, *T. indica*, and *Ceilba pentandrat, which* are well-known for their hypoglycemic properties. Therefore, the green synthesis methods are an attractive option. Green nanotechnology refers to the use of nanotechnology to enhance the environmental sustainability of processes producing negative externalities [7]. It includes making and using nano-products in support of sustainability. Biologically developed chemicals are used in these methods, which are not harmful for the environment [8]. Green nanotechnology has two goals: producing nanomaterials and products without harming the environment or human health, and producing nano-products that provide solutions to environmental problems [9]. Due to the wide range of applications of NPs, researchers, including biologists, chemists, physicists, and engineers, are working in this fascinating area [10]. Green synthesis has many advantages compared to chemical and physical methods; it is non-toxic, pollution-free, environmentally friendly, economical, and more sustainable [11,12]. Therefore, accessing green principles offers a high degree of safety, eco-friendliness, and cost-effectiveness. On green pathways, nanoparticles can be fabricated using natural compounds extracted from various biomass precursors, such as bacteria, fungi, biomolecules, and plant extracts [13]. The most important feature of the biogenic approach is the utilization of biologically reducing and capping agents to replace toxic chemicals. This alternation makes the biosynthetic method environmentally friendly, benign, and inexpensive [14].

The AgNPs in this study were developed under controlled conditions following parameters such as temperature, pressure, and reactant concentrations to obtain stable, spherical, and small-sized nanoparticles [15,16]. AgNPs have superiorities over other nanoparticles due to their outstanding properties such as small size, good conductivity, chemical stability, catalytic activity, optical, thermal, high electrical conductivity, and biological properties [17,18]. Moreover, the *Azadirachta indica* seed extract used in this study contains natural compounds such as Azadarachtin, Nimbin, and Nimbidin that have synergistic effects on AgNPs and enhance their anti-diabetic potentials [19,20]. A pH of 7 was the best to ensure the reduction of Ag+ to Ag0 during AgNPs production, and the greatest abundance of synthesized nanoparticles was obtained at pH 7–9. Several studies have shown that the production rate of AgNPs increases as the pH increases. Furthermore, AgNPs were almost spherical at higher pH values, and setting the pH at 8 substantially enhanced the reaction rate [21]. Temperature is one of the most important parameters that affects the size and morphology of biosynthesized AgNPs. Numerous studies have confirmed that the dimensions of AgNPs decrease as the reaction temperature increases, resulting in a change in their morphology [22].

The absorption intensity increased as the incubation time increased, owing to an increase in the amount of AgNPs produced. AgNPs have also been generated using Origanum vulgare L. extract, and the yield of nanoparticles increased with an increasing reaction time up to 3 h [23]. Song et al., 2022 [24] reported that absorbance increased when light intensity increased. Thus, it is expected that under sunlight, the reduction process of Ag+ ions can be completed within a few minutes, whereas the reaction requires a longer duration in the dark. Increasing the plant extract concentration in the reaction mixture can increase the absorbance intensity [25]. When using high extract concentrations, biomolecules act as reducing agents and cover the nanoparticle surfaces, preventing them from aggregating and increasing their stability. Due to their peculiar properties, they have been used for several applications, including as anti-bacterial agents, in industrial, household, and healthcare-related products, in consumer products, medical device coatings, optical sensors, and cosmetics, in the pharmaceutical industry, the food industry, in diagnostics, orthopedics, drug delivery, and as anti-cancer agents, and have ultimately enhanced the tumor-killing effects of anti-cancer drugs [26,27]. It has been shown that silver nanoparticles have anti-diabetic potential. Recent research in the field of plant-based nanomedicine has demonstrated that biosynthesized nanoparticles are more effective than crude extracts [28,29]. They have advantages, including increased surface area, solubility, and healing capacity. The synthesis of Ag nanoparticles using plants has several advantages compared to other biosynthesized nanoparticles, which is why the use of extract has received greater attention [30]. Moreover, the application of biomolecules as reducing, stabilizing, and capping agents rather than costly toxic chemicals makes the biosynthesis of nanoparticles an efficient process [31].

Several resources, such as pollens, polyoxometalates, irradiations, and polysaccharides, are employed for the environment-friendly biosynthesis of AgNPs [32]. The green synthesized silver nanoparticles have many applications, such as the breakdown of harmful pollutants, water purification, food preservation, and the production of nano-insecticides, nano-pesticides, and cosmetics [33]. Due to their bioactive nature, the scientific research community is now evaluating them as a new active mediator for the curing of diabetes mellitus [34]. Sharifi et al. synthesized the AgNPs through a green process and evaluated their anti-oxidant, anti-bacterial, and anti-inflammatory activities [35]. Jini and Sharmila have carried out the AgNPs synthesis through the green method and employed them as plant-mediated medicine for the management of diabetes [36]. Nagaraja et al. investigated the green synthesized AgNPs of leaf extract and stated that the particles act as an excellent anti-diabetic candidate [37]. Vinodhini et al. have biosynthesized AgNPs using *Allium fistulosum*, *Tabernaemontana divaricate*, and *Basella alba* extracts. The synthesized AgNPs exhibited high anti-oxidant and anti-diabetic activities [38]. By using *Acacia nilotica* extract, Zubair et al. sustainably created silver nanoparticles. They investigated the anticancer and anti-diabetic properties of the AgNPs [39]. Kaliammal et al. used an extract of *Zephyranthes candida* flower for the synthesis of AgNPs. Their results confirmed that the particles showed anti-diabetic, anti-inflammatory, anti-oxidant, and anti-cancer activities [40]. Badmus et al. synthesized the AgNPs using *Annona muricata* aqueous leaf extract. They found that the biosynthesized particles showed cytotoxicity in human keratinocyte cells (HaCaT) as well as in vitro anti-diabetic, antioxidant, lipid peroxidation inhibition, and anti-bacterial activities [41]. Das et al. (2021) biofabricated the AgNPs through green synthesis using *Dregea volubilis* flowers and established better anti-oxidant, anti-diabetic, and anti-bacterial activities [42]. Thirumal S. and Sivakumar used *Cassia auriculata* leaf extract for the green preparation of AgNPs and demonstrated their potent anti-diabetic activity [43]. Yarrappagaari et al. biosynthesized the AgNPs from the aqueous extract of *Cleome viscosa* and evaluated them for anti-bacterial, antioxidant, and anti-diabetic activities [44]. Sathiyaseelan et al. studied the fungus chitosan (FCS)-enclosed *Gynura procumbens* (GP) biosynthesized silver nanoparticles (GP-AgNPs) and found them to be excellent candidates for antibacterial and diabetic-associated enzyme inhibitory activities [45]. Similar studies reported AgNPs for their anti-diabetic, anti-cancer, anti-bacterial, and anti-inflammatory capacities [46]. The previous literature survey clearly indicates that various researchers have biosynthesized the AgNPs by taking different parts of the plant and evaluating their different biological activities.

According to previous literature [47], if the natural compounds coating the nanoparticles are themselves anti-diabetic in nature, a synergistic anti-diabetic potential of the final nanomaterial is observed [48], and *Azadirachta indica* seeds contain the most potent anti-diabetic compounds, such as Azadarachtin, Nimbin, Nimbidin, etc., so that must reveal high anti-diabetic potential; therefore, *Azadirachta indica* seeds extract-mediated AgNPs are better than those AgNPs reported earlier in the literature [49,50]. Considering the vast potentiality of plants as sources, this work aims to investigate the use of *Azadirachta indica* seed extract for the biosynthesis of AgNPs, and the synthesized AgNPs were pragmatically characterized and investigated for their anti-diabetic potential. To the best of our knowledge, this research will represent the first reference to the use of *A. indica* seed extract for the green synthesis of silver nanoparticles and anti-diabetic activities.

## 2. Materials and Methods

### 2.1. Extract Preparation from Azadirachta indica Seeds

For the preparation of *A. indica* seed extract, the method of Hameed et al. [51] was followed. *Azadirachta indica* (Neem) seeds were purchased from an herbal store, identified, and specimens placed in the herbarium of the Department of Botany, Abdul Wali Khan University Mardan (AWKUM), with an accession number of AWKUM. Bot. 425.1.20. *Azadirachta indica* seeds were rinsed gently with clean water, dried, and crushed to powder using a plant grinder (Panasonic Model MX-AC210, Osaka, Japan). For the preparation of methanolic extract, 1 kg powder of *Azadirachta indica* was incubated in 2000 mL of 100% methanol (used as solvent) and stored at room temperature (25 ± 3 °C) for 5 days. After filtration, the solution was rotary evaporated at 48 °C under reduced pressure and used for the biosynthesis of AI-AgNPs.

### 2.2. Green Synthesizing of Azadirachta indica Seeds-Mediated Silver Nanoparticles (AI-AgNPs)

**Figure 1:**
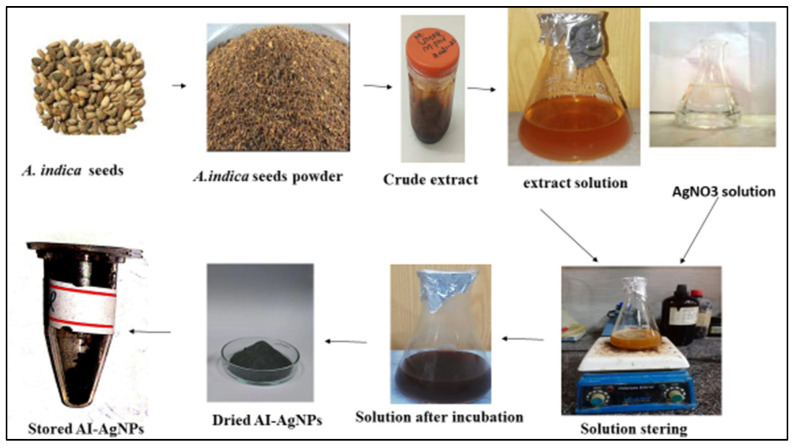
Green Synthesis of AI-AgNPs.

For the biosynthesis of AgNPs, the protocol of [52] was followed: a 100-mL solution of 1 mM AgNO_3_ was prepared in a conical flask, and 20 mg of the *A. indica* seed extract was added with continuous stirring on a hot plate below the boiling point (70 °C) for 4 h. The AgNPs solution was then exposed to light for the process of reduction. The green synthesis of the AI-AgNPs was observed by the changing color of the solutions, which was recorded at regular intervals. The reaction was set to continue for 24 h, and then centrifugation was performed for 30 min at 10,000 rpm. The supernatant was discarded after centrifugation, and the pellet was washed three times with double-distilled water. This was followed by centrifugation to remove any remaining free compounds and obtain pure nanoparticles that were used for anti-diabetic activities. A total of 0.017 g of precursor (AgNO_3_), which can theoretically yield 0.011 g of silver nanoparticles, and in this study, 0.015 g was obtained, which means the obtained efficiency is very good. A slight increase of 0.004 g in the weight of the obtained AgNPs is due to the coated organic layer on the surface of these particles. For further use, the precipitated AI-AgNPs were lyophilized and stored in a dry and cool place. Figure 1 represents the biosynthesis of AI-AgNPs.

### 2.3. Characterization of the Green Synthesized AI-AgNPs

The biosynthesized AI-AgNPs were examined for their physical, morphological, and chemical characteristics using different techniques, like UV-VIS spectroscopy, FTIR, SEM, and XRD.

#### 2.3.1. Visible Observation

According to literature studies, silver nanoparticle solutions have a dark brown or dark reddish color [53]. The color of the *A. Indica* solution before the addition of AgNO_3_ was yellowish, but after its treatment with AgNO_3_, it changed to dark brown, which indicated the formation of AgNPs. (Figure 1) This color change is due to the property of quantum confinement, which is a size-dependent property of nanoparticles that affects the optical properties of the nanoparticles.

#### 2.3.2. UV-Visible Spectral Analysis of AI-AgNPs

Using a UV-VIS spectrophotometer (Multiskan GO; Thermo Scientific, Waltham, MA, USA), the absorption spectra of the aqueous solution with dried 80 µg per mL silver nanoparticles were recorded at a 2-nm resolution between 200 and 800 nm to investigate how the light affected the green synthesis of AI-AgNPs. The experiment was repeated three times, and the absorption was measured at wavelengths between 200 and 800 nm with a 2-nm difference at a 1 h interval and recorded.

#### 2.3.3. FTIR Analysis of AI-AgNPs

To identify distinct functional groups in the green synthesized AI-AgNPs, FTIR (IRTracer-100, Shimadzu, Kyoto, Japan) was used at wavelengths from 4000 to 500 cm^−1^ and the corresponding data were recorded.

#### 2.3.4. X-ray Diffraction Technique (XRD) Analysis of AI-AgNPs

XRD was used to examine the purity and crystalline makeup of the biosynthesized AI-AgNPs following the method of [54] using an X-ray diffractometer (Model-D8 Advance, Bruker, Bremen, Germany).

#### 2.3.5. Scanning Electron Microscopy (SEM)

A high-resolution image of the AI-AgNPs was produced by SEM (Hitachi, S-4300SE, Tokyo, Japan), which includes details about their size, shape, composition, electrical conductivity, topography, and other characteristics.

### 2.4. In Vitro Anti-Diabetic Potential

#### 2.4.1. Assay for Uptake of Glucose by Yeast Cells

The glucose uptake by yeast cells experiment was conducted using a slightly modified method of Rehman et al. [55]. AI-AgNPs in various concentrations (10 µg/mL to 80 µg/mL) were combined with 1 mL of 5 mM glucose solutions, and the reaction solution was then incubated for 10 min at 37 °C. The final reaction was started by adding a 10%/V suspension of yeast. After being vortexed, the reaction mixture was kept for 60 min at 37 °C, then centrifuged for 5 min at 3800 rpm. The supernatant amount of glucose was determined using a spectrophotometer (UV 5100B spectrophotometer). First, the absorbance value of the control was found at 520 nm wavelength using the spectrophotometer, then the absorbance value of the test samples was determined, and the process was repeated in triplicate. The following formula was used to compute the uptake of glucose by yeast cells:

$$\%\;Glucose\;uptake=\frac{Absorbance\;Control-Absorbance\;Test\;sample\;}{\begin{array}{c} Absorbance\;Control \end{array}}\times 100$$

#### 2.4.2. Glucose Adsorption Assay

The glucose adsorption method was used to evaluate the ability of AI-AgNPs to adsorb glucose by following the method of [56]. 1 g of the AI-AgNPs was combined with a 100 mL solution of glucose (5–30 mM glucose). The final mixture was incubated in a shaker at room temperature for up to 6 h before being centrifuged for 20 min at 4800 rpm. The absorbance values of G1 (glucose concentration before reaction, using a spectrophotometer), then the absorbance value and G6 (after 6 h concentration of glucose) were determined at 520 nm wavelength. Using the following formula, the % glucose adsorption was calculated.

$$Bounded\;Glucose=\frac{G1-G6}{Sample\;weight}\times volume\;of\;sample$$

G1—Glucose concentration before reaction.

G6—After 6 h concentration of glucose.

#### 2.4.3. Alpha (α) Amylase Inhibition Assay

The alpha bonds of polysaccharides are hydrolyzed by the enzyme alpha-amylase to create glucose and maltose. This process of the alpha-amylase enzyme elevates blood sugar levels, and its inhibition will cause blood sugar levels to drop. According to the procedure described by [57], a reaction mixture containing porcine pancreatic amylase (500 µL) in phosphate buffer, a standard drug (250 µL), and AI-AgNPs was prepared and kept at 37 °C for 20 min. After that, 250 µL of PBS (100 mM) and 1% starch were added to the mixture and kept at 37 °C for 60 min. In the last step, 1 mL of a color reagent, di-nitro-salicylate was added to the solution and heated for 10 min. The optical density was determined at 540 nm. Using the formula below, the percentage of inhibition was calculated.

$$\%\;Inhibition=\frac{Control\;Absorbance-sample\;Absorbance\;}{Absorbance\;Control}\times 100$$

### 2.5. Analysis of In Vivo Antidiabetic Potentials

#### 2.5.1. Experimental Animals and Conditions

Adult BALB/C mice weighing 25–30 g and 5 weeks old were bought from the Veterinary Research Institute Peshawar. The animals were kept in controlled environments (22 °C, 60% humidity, 12 h of light and darkness), provided with standard food, and provided with free access to water. All the experiments were carried out strictly in accordance with the guidelines of the National Research Council for the handling and use of lab animals. Before starting the experiments, approval of the protocol was obtained from the Ethical Committee of the Department of Zoology, Abdul Wali Khan University, Mardan, Pakistan.

Group I: Normal Control (0.1 M Citrate Buffer, 0.5 mL, and pH 4.5);

Group II: Diabetic Control (Streptozotocin 50 mg/kg body weight);

Group III: Diabetic mice administered with AI-AgNPs (10 mg/kg body weight);

Group IV: Diabetic mice administered with AI-AgNPs (20 mg/kg body weight);

Group IV: Diabetic mice administered with AI-AgNPs (30 mg/kg body weight);

Group V: Diabetic mice administered with AI-AgNPs (40 mg/kg body weight);

#### 2.5.2. Induction of Diabetes in Mice by Streptozotocin

The mice were starved overnight before administering one dose (50 mg/kg body weight) of streptozotocin (STZ) was injected intraperitoneally into the diabetic groups, whereas citrate buffer alone was administered to the control group. The diabetes was confirmed by estimating the blood sugar after 72 h of STZ injection. Mice having a blood sugar of more than 120 mg/dl were classified as diabetic and employed in vivo experiments.

#### 2.5.3. Blood Glucose Level of Experimental Mice

The experimental mice were starved for 12 to 16 h, blood samples were taken by the tail vein rupturing method, and the blood sugar was estimated using a one-touch glucometer (Life Scan, Inc., Milpitas, CA, USA). The glucose levels were observed on the 1st, 5th, 10th, 15th, 20th, 25th, and 30th days.

#### 2.5.4. Histopathological Study of the Pancreas and Liver of Experimental Mice

The pancreas and liver were separated, and they were continually cleaned with phosphate-buffered saline. The tissue samples were subsequently fixed in 10% formalin and subjected to standard histopathology procedures. The sample tissues were immersed in wax and subsequently cut into 5μm-thick slices. Hematoxylin and eosin stain were used to stain the sections, and the morphology of the tissues was observed under the microscope.

#### 2.5.5. Statistical Analysis

The results were expressed as mean ± SD using GraphPad Prism version 9. For statistical analysis of the data group, the mean was compared using a one-way analysis of variance (ANOVA). In addition, *p* < 0.05 was considered to be statistically significant.

## 3. Results

### 3.1. Visible Observation

**Figure 2:**
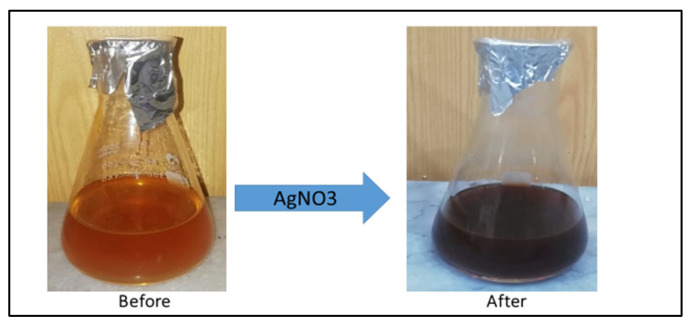
Color change of *A. indica* seed extract solution before and after the addition of AgNO_3_.

The color of the A. Indica solution before the addition of AgNO_3_ was yellowish, but after its treatment with AgNO_3_, it changed to dark brown, which indicated the formation of AgNPs (Figure 2). This color change is due to the property of quantum confinement, which is a size-dependent property of nanoparticles that affects the optical properties of the nanoparticles.

### 3.2. UV-Visible and Bandgap Energy Analysis of AI-AgNPs

**Figure 3:**
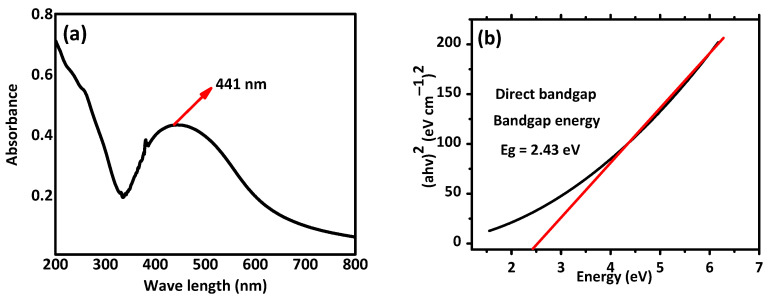
(**a**) UV-visible. (**b**) Tauc plots for bandgap energy analysis of AI-AgNPs. Plot of (αhν)^2^ against (hν) results in a straight line, which explains that the edge of absorption is due to the direct transition (*n* = 1 for direct transition). The optical band gap (Eg) is indicated with the intercept of a straight line.

UV-visible analysis and band energy values for the synthesized nanoparticles are presented in Figure 3. Silver nanoparticles have a peak UV absorption wavelength between 400 and 450 nm. The synthesis of AI-AgNPs in the *A. indica* seed extract is indicated by the UV-visible density peaks of the *A. indica* UV-Vis spectra at about 441 nm shown in Figure 3a. The process of surface plasmon resonance, which results from stimulation of the surface plasmons that exist on the external surface of the AI-AgNPs and which is stimulated due to the applied electromagnetic field, is what causes the peak at 380 nm to appear [58]. The below Tauc plot equation was used to obtain the bandgap energy value for the produced nanoparticles shown in Figure 3b.

(αhν)^γ^ = A(hν − Eg) (1)

where as α is the coefficient of absorption, h is the planks constant, ν is the frequency of photons, A is the proportionality constant, γ = electronic transition, and Eg is bandgap energy depending on the transition and can have values of 2, 1/2, 2/3, or 1/3. Tauc plots for AgNPs are presented in Figure 3b. The plot of (αhν)^2^ against (hν) results in a straight line, which explains that the edge of absorption is due to the direct transition (*n* = 1 for direct transition). The optical band gap (Eg) is indicated by the intercept of a straight line. The biosynthesized nanoparticles’ direct bandgap energy is Eg = 2.43 eV.

### 3.3. SEM Analysis of AI-AgNPs

**Figure 4:**
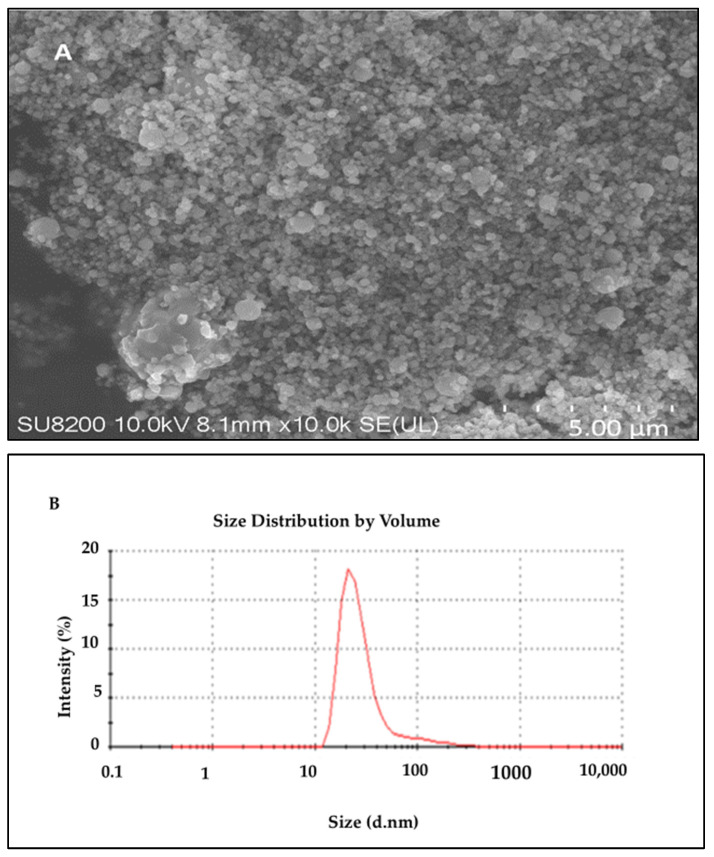
(**A**) SEM results of *A. indica-*mediated silver nanoparticles; (**B**) particle size analysis of AI-AgNPs using the DLS image system.

To evaluate the morphology of the biosynthesized AI-AgNPs, the scanning electron microscope (SEM; JEOL JSM-7001F) was used. Figure 4A indicates from the SEM result of the biosynthesized nanoparticles that the particles are evenly and uniformly distributed and have a spherical shape at the nanoscale. Some agglomerations of the synthesized nanoparticles were seen because of the plant extract. However, there are also various plant extract components that function as stabilizing and capping agents, reducing the aggregation of the particles. Agglomeration led to collecting the nanoparticles in ordinary shapes, mostly by physical bond, due to the nature of the solvent used. We used biological sources with organic moieties on their surfaces and functional groups that interact with each other and cause agglomeration. There are many ways to de-agglomerate nanoparticles, such as sonication, ultrasound, isopropyl storage, heat, electrostatic charge, etc. We have overcome this issue by sonicating AgNPs before using them for biological activities. The coated layer of organic moieties on the silver nanoparticles gives them stability, and as a result, the nanoparticles are stable and mostly dispersed. The size distribution histogram of dynamic light scattering (DLS) indicates that the average size of these silver nanoparticles is 34.43 nm. Figure 4 shows the DLS pattern of the suspension of AI-AgNPs.

### 3.4. Fourier Transform Infrared Spectrophotometry (FTIR) and X-Ray Diffraction (XRD) Analysis

**Figure 5:**
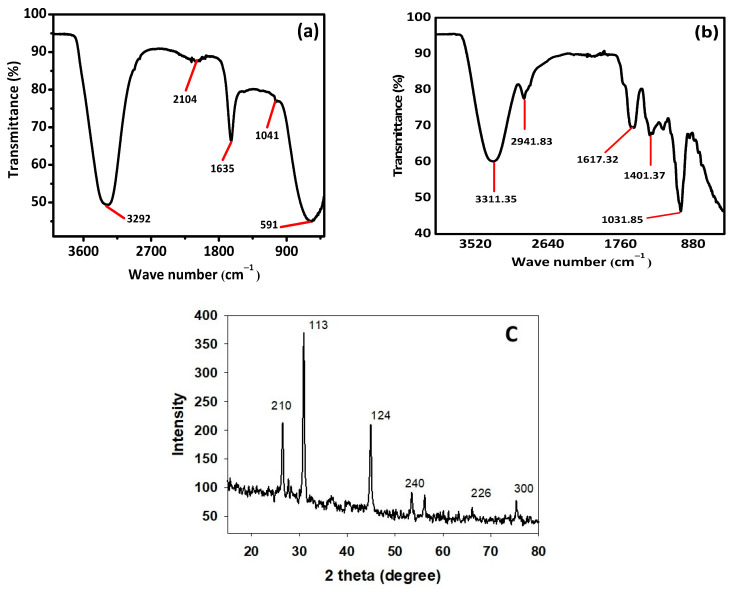
(**a**) FTIR of pure *A. indica*, (**b**) *A. indica-*mediated AgNPs, and (**c**) XRD spectrum of *A. indica-*mediated AgNPs.

FTIR and XRD analysis were performed for structural identification, crystallinity, and phase shifts using an FTIR spectrophotometer (Spectrum Two TM FT-IR Spectrometer; PerkinElmer, Waltham, MA, USA) at different wavelengths ranging from 200 to 4000 cm^−1^. Figure 5a,b shows the FTIR results of the pure *A. indica* and *A. indica-*mediated AgNPs. The extra band observed at 591 cm^−1^ corresponds to the Ag nanoparticles that appeared in *A. indica-*mediated AgNPs shown in Figure 5b. The peak at 1401.37 cm^−1^ may be due to the (C-O and C-H) bending vibrations of the *A. indica plant.* The stretching vibration of the C-O functional group of alcohol, ester, ether, or carboxylic acid is shown by the peaks at 1031.85 cm^−1^ and 1041 cm^−1^, respectively. The peak at 1617.32 cm^−1^ and 1635 cm^−1^ might be caused by the C=O stretching vibration of alkenes, primary amines (N-H bending vibration), and amides (N-H bending and C=O stretching vibration), as well as the functional groups of aldehydes and ketones. The stretch at 2104 cm^−1^ is due to the C≡C of the alkene. The OH stretching vibration of the phenol causes the peaks at 3292 cm^−1^ and 3311.35 cm^−1^ respectively. Additionally, the presence of OH and C=O groups suggests that flavanones or terpenoids have been adsorbed on the surface of nanoparticles. Connections via π-electrons in the carbonyl groups may be liable for the reduction of Ag ions to AgNPs as well as for stability and as a capping agent. The presence of various functional groups in Figure 5b demonstrated the successful green synthesis of *A. indica*-mediated AgNPs.

X-ray diffraction (XRD) is a popular analytical technique that has been used for the analysis of both molecular and crystal structures, qualitative identification of various compounds, quantitative resolution of chemical species, measuring the degree of crystallinity, isomorphous substitutions, particle sizes, etc. When X-ray light reflects on any crystal, it leads to the formation of many diffraction patterns, and the patterns reflect the physico-chemical characteristics of the crystal structures.

The XRD result given in Figure 5c shows that there are four separate diffraction peaks in the 2θ range of 10° to 80°. The peaks seen at 2θ angles of 26.23°, 30.66°, 44.56°, 56.22°, 66.08°, and 75.35° correspond to the 210, 113, 124, 240, 226, and 300, planes correspondingly, which were indexed for a silver face-centered cube of (JCPDS file no. 04-0783).

The result clearly explains that the biosynthesized AI-AgNPs were crystalline. These findings support the presence of face-centered cubic AI-AgNPs. The unassigned peaks might have resulted from the plant extract-dependent capping agent involved in the stabilization of AgNPs, and the average size of the AgNPs was 34.43 nm.

### 3.5. Effects of AI-AgNPs and Crude Extract on the Uptake of Glucose by the Yeast Cells

**Figure 6:**
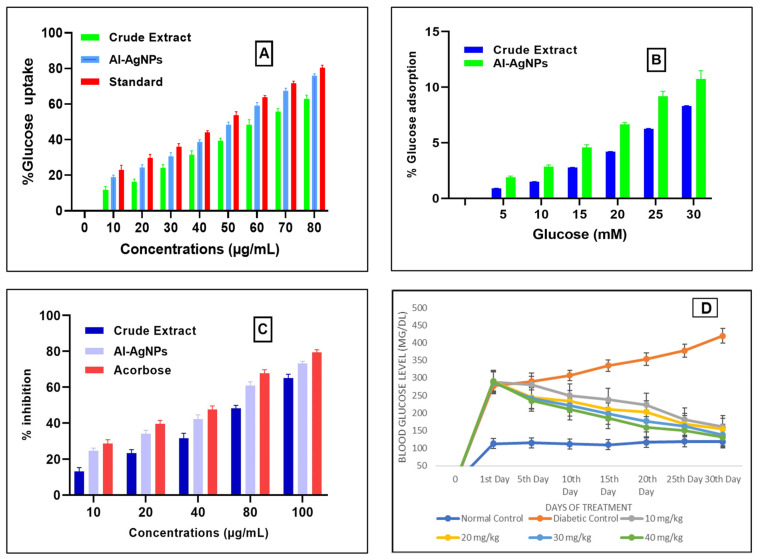
(**A**) Glucose uptake by yeast cells; (**B**) % Glucose adsorption; (**C**) inhibition of alpha-amylase; (**D**) blood glucose level of mice using *A. indica-*mediated AgNPs.

Uptake of glucose by the yeast cells at different concentrations like 10, 20, 30, 40, 50, 60, 70, and 80 µg/mL of AI-AgNPs and crude extract were determined as AI-AgNPs 18 ± 2.64%, 23 ± 2.082%, 29 ± 1.692%, 37 ± 1.00%, 48 ± 2.82%, 57 ± 0.854%, 66 ± 1.311%, and 75 ± 1.528%, respectively, whereas crude extract was determined as 11.804 ± 1.0%, 16 ± 1.36%, 24 ± 2.03%, 31.63 ± 2.98%, 39.5 ± 1.36%, 48.467 ± 2.73%, 55.01 ± 2.015%, and 63 ± 2.5%, respectively, Acarbose was used as a standard drug in the same concentration as AI-AgNPs, and crude extract was taken. The results showed 21 ± 1%, 28 ± 1.528%, 34 ± 2.582%, 43 ± 1.206%, 52 ± 1.58%, 63 ± 1.637%, 70 ± 1.528%, and 80 ± 1.20% glucose uptake, respectively, sowing significant result (*p*-value ≤ 0.05) indicated in Figure 6A.

### 3.6. Analysis of Glucose Adsorption by A. indica Seed Extract-Mediated AgNPs and Crude Extract

Figure 6B shows the glucose adsorption activity of the green synthesized AI-AgNPs and crude extract at various concentrations of glucose that are 5, 10, 15, 20, 25, and 30 mM. AI-AgNPs showed 1.8 ± 0.62%, 2.88 ± 0.06%, 4.33 ± 0.153%, 6.56 ± 0.50%, 8.95 ± 1.00%, and 10.65 ± 1.58% adsorption of glucose, respectively, and crude extract showed 0.9 ± 0.02%, 1.5 ± 0.032%, 2.77 ± 0.015%, 4.200 ± 0.20%, 6.25 ± 0.04%, and 8.32 ± 0.258% adsorption of glucose, respectively. The results indicate that this effect is not just due to the adsorption of glucose but also to the uptake of glucose. Because the adsorption of glucose depends on the concentration of glucose, as the glucose concentration increases, the adsorption of glucose also increases, and glucose uptake in yeast cells depends on the concentration of AI-AgNPs and crude extract.

### 3.7. Impact of AI-AgNPs and Crude Extract on Inhibition of α-Amylase

The alpha-amylase inhibitory effect of AI-AgNPs (10–100 µg/Ml) was determined for the prepared AI-AgNPs and crude extract, as shown in Figure 6C. The percent inhibitory values of AI-AgNPs at 10, 20, 40, 80, 100µg/mL were noted as 23.7 ± 1.4%, 34 ± 1.99%, 42.13 ± 2.44%, 60.92 ± 2.00%, and 73.85 ± 1.114%, respectively, with an IC50 value of 48.26 µg/mL and that of crude extract. The percent inhibitory values of crude extract at 10, 20, 40, 80, and 100µg/mL were noted as 13.7 ± 2.15%, 23 ± 2.67%, 31.500 ± 2.78%, 48.62 ± 2.167%, and 65.85 ± 2.101%, respectively, with an IC50 value of 68.37 µg/mL. Whereas acarbose, taken as a standard drug, showed % inhibition at 10, 20, 40, 80, and 100 µg/mL as 27 ± 2.082%, 39.56 ± 1.913, 47.6 ± 1.97, 67.66 ± 2.086, 10 µg/mL, and 79.33 ± 1.528, respectively.

### 3.8. Analysis of Blood Glucose Level of Experimental Mice

Administration of STZ resulted in increased blood glucose levels, which were reversed by treating diabetic mice with 10 to 40 mg/kg body weight of AI-AgNPs for 30 days, resulting in a significant decrease in hyperglycemia as shown in Figure 6D. It is clear from this figure that there was a significant increase in blood glucose levels (277 ± 5.1 to 420 ± 22.03 mg/dL) in the diabetic control group. The normal control group did not show a significant increase in blood glucose level (113 ± 3.5 to 118 ± 4.72 mg/dL). Groups treated with AgNPs at doses of 10, 20, 30, and 40 mg/kg, respectively, showed a significant decrease (*p* < 0.05) in glucose level (288 ± 5.0 to 160 ± 5.54 mg/dL, 290 ± 2.5 to 154 ± 5.033 mg/dL, 287 ± 4.2 to 138 ± 7.50 mg/dL, 290 ± 8.3 to 131 ± 7.024 mg/dL), respectively.

### 3.9. Histological Study of Mice Pancreas

**Figure 7:**
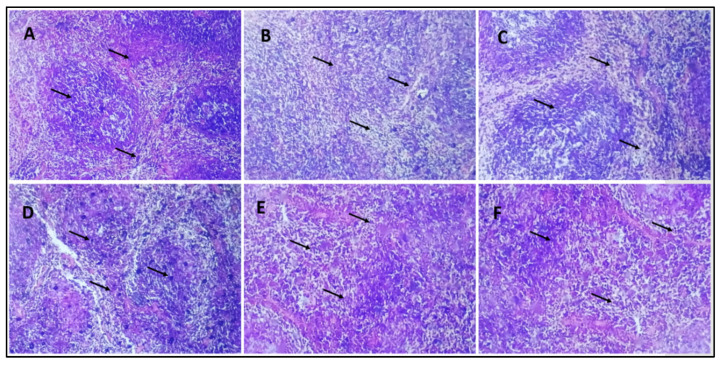
Histology section of the pancreas (**A**) Group–I: in normal control small lobules are densely packed with acinar cells (indicated by the arrow) (**B**) Group-II: in diabetic control β-cells of pancreas are almost entirely damaged (indicated by the arrow) (**C**) Group-III: treated with 10 mg/kg b/w AI-AgNPs; shown initial restoration of islets of the pancreas (indicated by the arrow) (**D**) Group-IV: treated with 20 mg/kg b/w AI-AgNPs; shows some restoration of islets of the pancreas (indicated by the arrow (**E**) Group-V: treated with 30 mg/kg b/w AI-AgNPs revealed moderate regeneration of islet cells (indicated by the arrow) (**F**) Group-VI: treated with 40 mg/kg b/w AI-AgNPs shown nearly normal shaped Islets of Langer-hans (indicated by the arrow).

In the histological studies of pancreas, the normal control group displayed the pancreas with normal anatomy, which shows that the exocrine component of pancreas is well organized into small lobules and is densely packed with acinar cells, and the pancreatic lobules are divided by septa into healthy intralobular and interlobular connective tissue (Figure 7A). Pathological abnormalities in both the exocrine and endocrine systems were seen in the pancreas of the diabetic control group. Little vacuoles [shown by an arrow] in the enlarged acinar cell can be seen nearly in all the acinar cells. β-cells in the pancreas of STZ-treated mice are almost entirely damaged (Figure 7B). The diabetic group receiving a dose of 10 mg/kg b/w AI-AgNPs displayed general architectural deformation of the pancreas. Acinar injury manifested by cytoplasmic vacuolation and cell atrophy was seen in most exocrine acini [indicated by the arrow] of the pancreas (Figure 7C). Diabetic mice treated with a dose of 20 mg/kg b/w AI-AgNPs showed some restoration of islets of the pancreas. The basal region of acinar cells has medium-sized vacuoles (Figure 7D). The diabetic group treated with a dose of 30 m g/kg b/w AI-AgNPs revealed regeneration of islet cells. Moreover, the tiny vacuoles in the basal region of the acinar cells were significantly smaller (Figure 7E). The diabetic mice treated with a dose of 40 mg/kg b/w AI-AgNPs displayed the nearly normal shape of Islets of Langerhans [indicated by the arrow]. The moderate atrophic alteration occurred in the acinar cells, and the distinction between the exocrine and endocrine sections of the pancreas improved (Figure 7F).

### 3.10. Histological Analysis of Mice Liver

**Figure 8:**
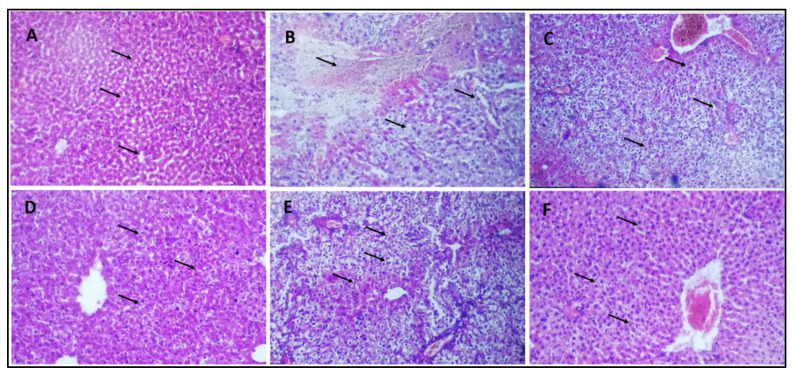
Histology sections of the liver. (**A**) Group–I: normal control; vacuoles and sinus are normal (indicated by arrow) (**B**) Group-II: diabetic control; Severe deterioration and necrosis are shown (indicated by arrow) (**C**) Group-III: treated with 10 mg/kg b/w AI-AgNPs; Some decrease occurred in deterioration and necrosis (indicated by arrow) (**D**) Group-IV: treated with 20 mg/kg b/w AI-AgNPs; moderate vacuoles and sinus regeneration are revealed (indicated by arrow) (**E**) Group-V: treated with 30 mg/kg b/w AI-AgNPs; Noticeable vacuoles and sinus regeneration occurred (indicated by arrow) (**F**) Group-VI: treated with 40 mg/kg b/w AI-AgNPs. Nearly normal vacuoles and sinus are shown (indicated by arrow).

The histology of the normal control group showed a typical normal histological picture of the liver (Figure 8A). The hepatocytes of the diabetic control group showed significant deterioration and necrosis in the liver (Figure 8B). Differences in the size of vacuoles of hepatocytes can be seen in the livers of mice treated with a 10 mg/kg b/w dose (Figure 8C). In the livers of mice treated with doses of 20 mg/kg b/w and 30 mg/kg b/w, there was a noticeable decrease in liver deterioration and necrosis of hepatocytes (Figure 8D,E). The diabetic group of mice treated with 40 mg/kg b/w showed a histological appearance that was comparable to the normal control group (Figure 8F).

## 4. Discussion

Nanotechnology has emerged as a leading technology in several sectors, with an ultimate application in agriculture, food, pharma, and biomedicine engineering. Nanoparticles, due to their small sizes, versatility, and readiness to couple with optical, textile, magnetic, electronic, mechanical, and chemical substances, are the candidates for novel applications in therapeutics, for example, anti-microbial, anti-oxidant, and cancer. In addition, nanoparticles have been extensively studied in the physical, chemical, and biological sciences [59]. Recently, several successful reports affirmed the production of these nanomaterials from natural sources such as plants and microbes. The biosynthesis of nanocomposites is a large-scale scientific domain with significant attention in biomedical applications due to their biocompatibility and multifunctional abilities [60].

Today’s most popular oral anti-diabetic medications rarely provide long-lasting glycemic control. To fill the gap, several medicinal plant extracts are deemed effective in lowering blood glucose levels and are administered as anti-diabetic drugs [61]. Several research studies have described the role of metals and their oxides, such as silver, vanadium, chromium, magnesium, and zinc oxides, in carbohydrate metabolism and the control of DMT2 [62]. The preparation of Ag nanoparticles via the green synthesis approach is effective due to the use of non-toxic phytochemicals and the absence of hazardous elements as found in the chemical method [63]. There are three basic categories of these techniques, including biological, physical, and chemical methods. The best method for synthesizing nanoparticles is the biological method due to its simple, nontoxic, and cost-effective nature [64].

The biosynthesis of AgNPs from plants is an easy procedure that includes the interaction of silver nitrate (AgNO_3_) with the biomolecule components of plant extracts [65]. Nanoparticles are formed primarily in three phases: an ion reduction reaction leads to cluster formation and then induces the growth of nanoparticles [66]. Each stage has unique characteristics depending on the reducing agent, its concentration, AgNO_3_, and pH. The presence of hydroxyl groups (OH) in plant biomolecules, such as amino acids, proteins, alkaloids, flavonoids, polyphenols, enzymes, tannins, carbohydrates, and saponins, is associated with the stabilization and reduction of silver ions (Ag+) to Ag0. Its further reduction to Ag + leads to the formation of silver nuclei, resulting in the production of AgNPs [67]. Considering the vast potential of plants as sources of medicinal compounds, this work aims to biosynthesize AgNPs using *Azadirachta indica* seed extract. The synthesized AI-AgNPs were pragmatically characterized and investigated for their anti-diabetic potential for the first time. To the best of my knowledge, this research will represent the first reference to the use of *A. indica* seed extract for the green synthesis of silver nanoparticles in Pakistan. According to literature [68], *A. indica* is thought to have insulin-like effects, which could help lower blood glucose levels by improving insulin sensitivity or acting as an insulin mimetic. It may protect pancreatic beta cells from damage that is responsible for insulin production. Preserving these cells is crucial for maintaining proper insulin levels. Components in neem may contribute to the reduction of blood glucose levels, helping in the management of diabetes [69]. Furthermore, synergistic effects between *A. indica* and silver nanoparticles exist [70]. As reported in the previous literature, if the natural compounds coating the nanoparticles are themselves anti-diabetic in nature, a synergistic anti-diabetic potential of the final nanomaterial is observed, and *Azadirachta indica* seeds contain the most potent compounds, such as Azadarachtin, Nimbin, Nimbidin, etc., so that must reveal high anti-diabetic potential [71].

Silver nanoparticles can be a potential source of insulin sensitization as they increase the cytosolic calcium ion concentration and activate AMPK by phosphorylating it via the CAMKKβ pathway in SH-SY5Y cells and in rats [72]. AMPK activation enhances the sensitivity towards insulin, and it could mediate the insulin by increasing its action [73]. Insulin binds to its receptor and activates the phosphorylation cascade from IRS1, which induces the transport of glucose into the cells [57]. Studies have shown that animal models lacking IRS1 developed hyperglycaemia, or Type 2 diabetes mellitus; hence, increasing the protein levels of IRS1 will ultimately reduce the hyperglycemia complications [74]. Silver nanoparticles lead to a reduction in blood glucose levels by increasing the IRS1 and GLUT2 expression levels. In addition, silver nanoparticles elevate the expression levels of insulin and its secretion [75]. Natural compounds acting as high reducing agents (Azadirachtin, Vepinin, Limbocidin, etc.) present in the *Azadirachta indica* seed extract strongly affect the size and size distribution of nanoparticles; the stronger the reductant present in the extract, the higher the reaction rate, resulting in the synthesis of nanoparticles with a smaller size [76]. At the same time, the particle size distribution of nanoparticles remains narrow. Reducing agents are essential in the fabrication of nanoparticles to enhance their biomedical functionality by reducing their toxicity and enhancing their biocompatibility and bioavailability in living cells. They prevent clusters or aggregates of nanoparticles, enhance their colloidal stability, and prevent the uncontrolled growth of nanoparticles (especially metal and metal oxide nanoparticles) [77].

People are interested in nanotechnology in the fields of physics, chemistry, biology, nanomedicine, and electronics as a result of the contemporary scientific period. Nanotechnology may create a wide range of nanoscale materials with at least one dimension, ranging in size from 1 to 100 nm, known as nanoparticles (NPs) [78]. Because of their capacity to be modified at a scale where characteristics can be controlled, nanomaterials have opened new areas of scientific and industrial innovation.

In the current study, silver nanoparticles were synthesized through a biological method using the extract of *Azadirachta indica* seeds, which are well known for their strong biological potential. The present investigation indicated that post-treatment improved the hyperglycemic condition of STZ-induced mice with AI-AgNPs. These nanoparticles were characterized by visual examination (Figure 2), ultraviolet–visible (UV–vis) spectrophotometry (Figure 3), scanning electron microscopy (SEM) (Figure 4), and Fourier transform infrared (FTIR) (Figure 5a), which are in line with [41]. The formation of AI-AgNPs was predominantly detected by the variation in color of the reaction mixture from light brown to dark brown after treatment with a 1 mM silver salt (AgNO_3_) solution. Several characterization techniques supported the successful production of NPs. The silver nanoparticles produced from *A. indica* showed a brown color change at an optimized time of 3 h. The maximum absorption was at 441 nm from the UV-visible analysis. Similar results were observed for silver nanoparticles in previous literature. Rajesh Kumar and Malarkodi [79] got an absorbance peak in the range of 400 to 450 nm when synthesized from different sources. The FTIR results of the pure *A. indica* and *A. indica-*mediated AgNPs. The extra band observed at 591 cm^−1^ corresponds to the Ag nanoparticles that appeared in *A. indica-*mediated AgNPs, as indicated in Figure 5b. The peak at 1401.37 cm^−1^ may be due to the (C-O and C-H) bending vibrations of the *A. indica plant.* The stretching vibration of the C-O functional group of alcohol, ester, ether, or carboxylic acid is shown by the peaks at 1031.85 cm^−1^ and 1041 cm^−1^, respectively. The peak at 1617.32 cm^−1^ and 1635 cm^−1^ might be caused by the C=O stretching vibration of alkenes, primary amines (N-H bending vibration), and amides (N-H bending and C=O stretching vibration), as well as the functional groups of aldehydes and ketones. The stretch at 2104 cm^−1^ is due to the C≡C of the alkene. The OH stretching vibration of the phenol causes the peaks at 3292 cm^−1^ and 3311.35 cm^−1^, respectively. Additionally, the presence of OH and C=O groups suggests that flavanones or terpenoids have been adsorbed on the surface of nanoparticles. Connections via π-electrons in the carbonyl groups may be liable for the reduction of Ag ions to AgNPs as well as for stability and as a capping agent. The presence of various functional groups in Figure 5b demonstrated the successful green synthesis of *A. indica*-mediated AgNPs [80,81]. The role of AgNPs in various metabolic diseases has been studied. In the present study, the AI-AgNPs were assessed for their anti-diabetic activity through in vitro studies using glucose uptake by yeast cells assays, glucose adsorption assays, and alpha-amylase inhibitory assays, as well as an in vivo study on STZ-induced diabetic mice. Glucose uptake by yeast cells at various concentrations of AI-AgNPs has determined good results (Figure 6A). These results were found to be good and in line with [77]. AI-AgNPs showed the highest activity (75 ± 1.528%), while crude extract showed (63 ± 2.5%) glucose uptake by yeast at 80 µg/mL. In the glucose adsorption assay, the highest activity of Al-AgNPs was 10.65 ± 1.58%, while crude extract showed 8.32 ± 0.258% at 30 mM, whereas in the alpha-amylase assay, Al-AgNPs exhibited the maximum activity of 73.85 ± 1.114% and crude extract 65.85 ± 2.101% at 100 µg/mL. These results were found to be similar to previous work [76].

Administration of STZ resulted in increased blood glucose levels; the treatment of AI-AgNPs has reversed the hyperglycemic condition of diabetic mice. AI-AgNPs (10 to 40 mg/kg b.w.) treatment for 30 days resulted in a significant decrease in blood glucose level (Figure 6D). It is clear from this figure that there was a significant increase in blood glucose levels (277–420 mg/dL) in the diabetic control group. The normal control group did not show a significant increase in blood glucose level (113–118 mg/dL). Groups treated with AgNPs at doses of 10, 20, 30, and 40 mg/kg, respectively, showed a significant decrease (*p* < 0.05) in glucose level (288–160, 290–154, 287–138, 290–131 mg/dL), respectively. Similar results were shown by [82,83].

In histopathological studies, the normal control group shows a normal architecture of the pancreas. The exocrine component forms a pancreas closely packed by acinar cells and arranged into small lobules. Pancreatic lobules are separated by intact intralocular and interlobular connective tissue septa, while the diabetic control group revealed pathological changes of both exocrine and endocrine components. The acinar cells were swollen, and small vacuoles were observed in almost all acinar cells. Islet β-cells are almost entirely lost in STZ-treated mice. Diabetic mice treated with different doses of AI-AgNPs revealed regeneration of islet cells. The small vacuoles in the basal area of acinar cells were also much smaller. The atrophic change of the acinar cells was less severe, and the border between the exocrine and endocrine portions became more distinct (Figure 7). These results are in line with [84,85,86,87,88,89].

Histological studies of the liver were carried out. Severe degeneration and necrosis in the hepatocytes were detected in the diabetic control group. Variations in the size of vacuoles were determined in the cytoplasm of hepatocytes in the 10 mg/kg b.w. AI-AgNPs-treated group. Degeneration and necrosis were found to be significantly reduced in the livers of mice in doses of 20 and 30 mg/kg b.w. of AI-AgNPs-treated groups. Similar histological appearance to the control group was found in the dose-40 mg/kg b.w. AI-AgNPs-treated group except for slight vacuolation and dilation of sinusoids (Figure 8). These results are in line with other mechanisms of interaction of nano Ag with cells that have been presented in the study [90], and various researchers have reported different approaches for diabetic medicine [91,92,93,94,95,96,97]. Considering the results obtained from anti-diabetic activity, green-synthesised AgNPs are good anti-diabetic agents. They may be crucial resources in the pharmacological and therapeutic domains for treating diabetes and other metabolic illnesses.

## 5. Conclusions

In this study, *Azadirachta indica-*conjugated silver nanoparticles were synthesized using their seed extract. The biosynthesized silver nanoparticles were proved to have excellent anti-diabetic potentials, which are due to the presence of natural compounds coating the nanoparticles, which are themselves anti-diabetic in nature. A synergistic anti-diabetic potential of the final nanomaterial was observed. Therefore, AgNPs produced by *A. indica* may be potentially utilized for the economical production of AgNPs for many pharmaceutical applications. To the best of my knowledge, this research will represent the first reference to the use of *A. indica* seed extract for the green synthesis of silver nanoparticles and their anti-diabetic activities in Pakistan.

## Data Availability

Data is contained within the article.
